# Morphological and molecular characterization of adults and larvae of *Crassicauda* spp*.* (Nematoda: Spirurida) from Mediterranean fin whales *Balaenoptera physalus* (Linnaeus, 1758)

**DOI:** 10.1016/j.ijppaw.2019.06.004

**Published:** 2019-06-07

**Authors:** F. Marcer, E. Negrisolo, G. Franzo, C. Tessarin, M. Pietrobelli, E. Marchiori

**Affiliations:** a Department of Animal Medicine, Production and Health, University of Padova, 35020, Legnaro (PD), Italy; b Department of Comparative Biomedicine and Food Science, University of Padova, 35020, Legnaro (PD), Italy

**Keywords:** Fin whale, *Balaenoptera physalus*, Nematoda, *Crassicauda*, ITS-2, *cox1*

## Abstract

*Crassicauda boopis* is known to infect the kidneys and vascular system of mysticetes included *Balaenoptera physalus* and has been recently reported in Mediterranean waters. Identification at the species level relies on the observation of morphological features of the adult parasites, but field conditions during necropsy and the massive reaction of the host's immune system often prevent optimal conservation of the extremities. Moreover, larval stages of *Crassicauda* have never been described and no sequences are available in public databases to help such identification. Adult and larvae of *Crassicauda* were isolated from four specimens of *B*. *physalus* and studied with morphological and molecular techniques. Specimens of *C*. *anthonyi*, *C*. *grampicola* and *Crassicauda* sp. isolated from *Ziphius cavirostris*, *Grampus griseus*, *Stenella coeruleoalba* and *Tursiops truncatus* respectively were studied as well. Sequences of nuclear markers 18S and ITS-2 and of mitochondrial gene *cox1* were obtained and phylogenetic relationships within the genus *Crassicauda* were analysed. Analysis of the ITS2 grouped the different species in accordance with morphological identification, as already evidenced in literature for other Spirurida. A higher intra-specific variability was observed for the *cox1* gene, for which two species (*C. grampicola* and *C. anthonyi*) did not appear as monophyletic in the tree. Well-developed non-attached larval specimens in the intestinal lumen of a whale calf were molecularly identified as *C*. *boopis*, allowing new insights on the life cycle of this species. This work broadens the genetic database on cetaceans parasites, allowing species identification even in challenging field conditions or in poor conservation of the samples; moreover, the first morphological description of *C*. *boopis* larvae is provided.

## Highlights

• *Crassicauda boopis*, *C. anthonyi*, *C. grampicola* and a *Crassicauda* sp. are molecularly studied here.
• First morphological descriptions of larval stages of *Crassicauda* are provided.
• Phylogenetic analyses show ITS-2 as the more efficient marker compared to *cox1* for identification.
• New insights on life cycle of *Crassicauda boopis* are described.

## Graphical abstract

**Figure:**
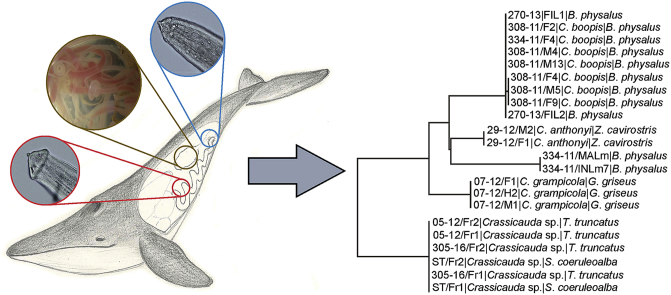

## 1 Introduction

Parasites of the order Spirurida are a diverse group of large nematodes affecting terrestrial and aquatic vertebrates. The genus *Crassicauda* Leiper and Atkinson, 1914 (Spirurida: Tetrameridae) infect different species of cetaceans, both toothed and baleen whales*.* Different organ tropism and consequent pathogenic impact are described among the members of this genus, whose localization spans over subcutaneous tissues, cranial sinuses and the urogenital system (Lambertsen, 1986; Geraci and St. Aubin, 1987; Jabbar et al., 2015). *Crassicauda boopis* Baylis (1920), with its large size and localization in the renal and circulatory systems, is considered one of the most pathogenic species in whales, similarly to *Crassicauda anthonyi* Chabaud, 1962in Cuvier's beaked whales (Dìaz-Delgado et al., 2016). Infections by *C. boopis* have been reported in fin whales (*Balaenoptera physalus*, Linnaeus, 1758), humpback (*Megaptera novaeangliae*, Borowski 1781) and blue whales (*Balaenoptera musculus*, Linnaeus, 1758) (Baylis, 1920; Cockrill, 1960; Lambertsen, 1992; Lempereur et al., 2017). Reports of this species have been provided from the North and South Pacific Ocean (Baylis, 1920; Rees, 1953; Margolis and Pike, 1955; Delyamure, 1955; Cockrill, 1960) and the North Atlantic Ocean (Hamilton, 1914, 1915; Lambertsen, 1985, 1986; Lempereur et al., 2017). Recently, *C. boopis* has been recorded in fin whales stranded along the coast of the Mediterranean Sea (Marcer et al., 2019) and nematodes tentatively classified as the same species have been observed in a fin whale stranded along Atlantic Spanish coasts, close to the Gibraltar strait (Fernàndez-Maldonado, 2018). Thrombosis of the renal veins and of the vena cava, abscessation and fibrosis of the kidneys are lesions commonly associated with the presence of cephalic portions of female nematodes within the lumen of the vena cava and with the presence of the body of the parasites within renal parenchyma. The impairment of blood supply to the kidney can finally result in congestive renal failure (Lambertsen, 1992).

Few hypotheses on the life cycle of *Crassicauda* spp. have been formulated (Lambertsen, 1992; Dìaz-Delgado et al., 2016). The presence of an intermediate or paratenic host is a reliable hypothesis, as marine spirurids have indirect cycles (Anderson, 2000). *Meganyctiphanes norvegica* (Crustacea: Euphasiacea) or other crustaceans included in rorquals diet could play a role in a hypothetical indirect life cycle of *C*. *boopis*, in which infective larvae would get into the host during filter-feeding. Nevertheless, a direct transmission whale-to-calf has been hypothesized during lactation, because of the presence of adult nematodes in the kidneys of waning calves (Lambertsen, 1986; Lempereur et al., 2017). Since no evidences of nematodes in the placenta or in the mammary tissue have ever been detected, the urine-contaminated milk is considered the most probable vehicle of infection from mother to calf. Based on the presence of vasculitis, sclerosis, and linear scars along mesenteric arteries and aortic walls, a larval migration from the intestine to the kidneys through vascular walls has been hypothesized for larvae of *C*. *boopis* (Lambertsen, 1992), like in *Crassicauda* sp. in Cuvier's beaked whales (Diàz-Delgado et al., 2016). However, these migrating immature specimens have never been isolated nor morphologically described in literature.

The identification of *Crassicauda* spp. relies on the observation of the morphometric features of cephalic and terminal fragments of the adult specimens. The remarkable body length of some species and the massive host reaction around the parasite often prevent the collection of intact specimens, making molecular methods useful alternatives for species identification. Recent phylogenetic studies have revealed *Crassicauda* species to be part of a clade comprised of members of the superfamily Acuarioidea (Jabbar et al., 2015; Diaz-Delgado et al., 2016; Lempereur et al., 2017), questioning all precedent morphological classifications which had placed the genus within the superfamily Habronematoidea, family Tetrameridae (Lambertsen, 1985). Nevertheless, current molecular characterization of *Crassicauda* species is limited and restricted to a few number of species and specific genes, which do not prove efficient in species discrimination. Broadening the genetic databases with sequences of other genes and from other species is essential for the study of species-discriminating markers and for solving phylogenetic issues.

The aim of this work is to: i) morphologically and molecularly characterize adult and larval stages of nematodes isolated from Mediterranean fin whales, ii) contribute to the genetic database of *Crassicauda* species and iii) acquire information about migration and localization of immature stages of *C*. *boopis* inside the final host.

In order to reach molecular identification, analysisof nuclear and mitochondrial markers was performed on different *Crassicauda* species to test the presence of molecular markers useful for species discrimination.

## 2 Materials and methods

### 2.1 Morphological characterization of nematodes collected from *B. physalus*

Portions of adult nematodes (N = 95 tails, 12 cephalic portions) were isolated from the vena cava, ureters and kidneys of three fin whales stranded along Italian Tyrrhenian coasts in the period 2008–2013 (Marcer et al., 2019). Three cephalic portions, 3 male tails and 3 female tails of these adult nematodes (whales #24-08, 334-11, 308-11) were used to achieve morphological identification (Table 1).

**Table 1:** Fin whales and specimens of nematode (larvae and adults) morphologically and molecularly (in brackets) analyzed in this study.

Species and ID	Site and year of stranding	Age (size)	CCC^a^	Sex	Adult nematodes				Nematode larvae		
					Female tails	Male tails	Heads	Internal fragments	Intestinal nodules	Mesenteric arteries	Intestinal lumen
Fin whale #24-08	Giannella (GR), 2008	Juvenile (13.4)	2	M	1	1	1	–	–		
Fin whale #334-11	Savona (SV), 2012 2011	Juvenile (10.0)	1–2	M	1 (1)	1	–	–	7 (1)	4 (1)	–
Fin whale #308-11	Capotesta (OT), 2011	Juvenile (10.78)	2	F	1 (3)	1 (3)	2	–	–		
Fin whale #270-13	Marciana (LI), 2013	Newborn (5.0)	2	F	–	–	–	–	–	–	9 (2)
Bottlenose dolphin #305-06	Porto Tolle (RO), 2006	Adult (1.90)	2	M	–	–	–	(2)	–	–	–
Cuvier's beaked whale #29-12	Calabria, 2012	–	4–5	M	1 (1)	1 (1)	4	–	–	–	–
Risso's dolphin #07-12	Bibione (VE), 2012	Adult (3.0)	2	F	2 (1)	2 (1)	2 (1)	–	–	–	–
Striped dolphin #05-12	Porto Garibaldi (FE), 2012	Adult (1.90)	2–3	F	–	–	–	(2)			
Bottlenose dolphin #60536	Aglientu (OT), 2014	–	–	–	–	–	–	(2)			

a CCC, Carcass condition code (*sensu*Geraci and Lounsbury, 2005). Size of the animals is given in meters.

Larvae of nematodes were isolated from two fin whales (*B. physalus*), stranded in the same geographical area. In particular, seven larvae were collected from nodules embedded in the intestinal wall (INL) and four included in the mesenteric artery walls (MAL) of one adult fin whale infected also by adult nematodes in the kidneys (whale #334-11); nine free larvae were collected from the intestinal lumen (FIL) of a whale calf (#270-13) in absence of any adult nematode (Table 1). All these larval specimens were used for morphological observations.

#### 2.1.1 Additional comparative material

Since few sequences of *Crassicauda* spp. are available in public genetic databases, specimens belonging to this genus, collected from cetaceans stranded along Italian coastline during the period 2006–2014, were also morphologically and molecularly characterized and included in this study (Table 1).

Specimens of adult nematodes isolated from the kidneys of a Cuvier's beaked whale (*Ziphius cavirostris*, G. Cuvier, 1823), and from the pterygoid sinuses of a Risso's dolphin (*Grampus griseus*, G. Cuvier, 1823), were morphologically identified as *C. anthonyi* (4 cephalic portions, 1 male tail and 1 female tail) and *C. grampicola*, Johnston and Mawson, 1941 (2 cephalic portions, 2 male tail and 2 female tail), respectively. One cephalic portion, one male tail and one female tail of both species were deposited at the Natural History Museum of London (NHMUK) (*C. anthonyi*: 2018.4.25.3–4; *C. grampicola*: 2018.4.25.1–2). Finally, fragments of *Crassicauda* sp. isolated from subcutaneous tissues of two bottlenose dolphins (*Tursiops truncatus*, Montagu, 1821) and of one striped dolphin (*Stenella coeruleoalba,* Meyen, 1833) were included in the study. Due to the lack of the cephalic and caudal extremities, these nematodes could not be identified to species level. Data of the hosts and nematodes analyzed in this study are listed in Supplementary Table 1.

All the collected nematodes (larvae and adults) were preserved in 70% ethanol. Adult parasites (cephalic and caudal ends) and the whole body of larvae, selected for morphological identification, were clarified in Amman's lactophenol solution and in 10% glycerol and 70% alcohol solution respectively. All samples were subsequently observed and measured with a light microscope (Olympus, ACH 40X-2) by NIS-Elements D software (Nikon).

### 2.2 Molecular analyses

DNA was isolated from the following specimens: 7 adult nematodes from fin whales; 4 larvae from mesenteric arteries and intestinal nodules and 3 larvae from the intestinal lumen of fin whales; 2 specimens of *C. anthonyi*; 3 specimens of *C. grampicola*; 6 fragments of *Crassicauda* sp. (Supplementary Table 1 and Table 1). Extraction was performed using NucleoSpin^®^ Tissue Kit (Macherey-Nagel, Germany), according to the manufacturer's instructions.

The small subunit (SSU; 18S) was amplified by polymerase chain reaction, using the primers G18S4–F (5′-GCTTGTCTCAAAGATTAAGCC-3′) and reverse 136-R (5′- TGATCCTTCTGCAGGTTCACCTAC-3′) (Nadler et al., 2007). The PCR for 18S region was performed in a 30 μl reaction volume, comprising 3 μl DNA, 2 mM MgCl_2_, 0.25 mM dNTPs (MBI Fermentas, Germany), 1X PCR buffer, 0.5 μM of each forward and reverse primer, 1U Platinum Taq DNA Polymerase (Invitrogen). Molecular biology grade water was added up to the final volume. Cycling conditions comprised an initial activation step at 94 °C for 2 min, followed by 35 cycles of 94° for 30 s, 58.7° for 30 s, 72 °C for 80 s, with a final extension step of 72° for 7 min. The PCR products were resolved through electrophoresis runs in 2% agarose gel with SYBR^®^ Safe DNA gel stain (Invitrogen™, USA). The amplicons of PCR (fragments of expected size 1700 bp) were directly sequenced at Macrogen (Macrogen Europe, the Netherland) using PCR primers. Additionally, a couple of internal primers were designed specifically with software Primer3 to facilitate sequencing the 18S fragment, i.e. forward primer 437F (5′-AACTAAGAACGGCCATGCAC-3′) and reverse 1279R (CTCTCGGCATGAGGAGGTAG-3′) (length of internal sequence: 842 bp).

The chromatograms quality was evaluated using the software ChromasPro version 2.4.3 (Technelysium Pty Ltd, Australia). The consensus sequences were assembled with the program SeqMan available in the DNAstar package. The consensus sequences were compared with the non-redundant data base available in the GeneBank^®^ database using the software BLAST (Altschul et al., 1990).

Further the samples were characterized through the amplification of the ITS-2 fragment of the rDNA and of the mt-*cox 1* gene region. Primers D (5′-GAGTCGATGAAGAACGCAG-3′) and reverse B1 (5′-GAATCCTGGTTAGTTTCTTTTCCT-3′) were used for the ITS-2 region (Traversa et al., 2004) and forward primer JB3 (5′-TTTTTTGGGCATCCTGAGGTTTAT-3′) and reverse JB4.5 (5′-TAAAGAAAGAACATAATGAAAATG-3′) were used for *cox 1* (Bowles et al., 1992).

The PCR for ITS-2 region was performed in a 30 μl reaction, comprising 3 μl DNA, 2.5 mM MgCl_2_, 0.25 mM dNTPs (MBI Fermentas, Germany), 1X PCR buffer, 0.5 μM of each forward and reverse primer, 1U Platinum Taq DNA Polymerase (Invitrogen). Molecular biology grade water was added up to the final volume. Cycling conditions comprised an initial activation step at 94 °C for 2 min, followed by 35 cycles of 94° for 30 s, 58° for 30 s, 72 °C for 30 s, with a final extension step of 72° for 5 min.

The PCR for *cox 1* region was performed in a 30 μl reaction volume, comprising 3 μl DNA, 2,5 mM MgCl_2_, 0.5 mM dNTPs (MBI Fermentas, Germany), 1X PCR buffer, 1.25 μM of each forward and reverse primer, 1U Platinum Taq DNA Polymerase (Invitrogen). Molecular biology grade water was added up to the final volume. Cycling conditions comprised an initial activation step at 95 °C for 2 min, followed by 35 cycles of 94° for 40 s, 50° for 30 s, 72 °C for 30 s, with a final extension step of 72° for 5 min.

The PCR products were resolved through electrophoresis runs in 2% agarose gel with SYBR^®^ Safe. The PCR products (fragments of expected size 510 bp and 710 bp for ITS-2 and *cox 1* respectively) were sequenced at Macrogen (Macrogen Europe, the Netherland) using the same PCR primer pairs and chromatograms processed as previously described.

### 2.3 Phylogenetic analysis

Independent data set including the 18S, *cox1* and *ITS2* sequences obtained in the present study as well as orthologous counterparts belonging to *Crassicauda* genus available in GenBank (*C. magna* KX354835 and KM233410; *C. boopis*: KY263809 and *C. giliakiana*: LC057236-37; LC057239-41; LC057243-46), were created in accordance to in accordance to (see results). When available, sequences obtained from members of the same superfamily were included as outgroup. The multiple alignments were performed with the program Muscle (Edgar, 2004) implemented in MEGA7 software (Kumar et al., 2016). When coding regions were considered (i.e. *cox1*), the alignment was performed at codon level. To guarantee phylogenetic trees reliability, distantly related outgroup sequences were maintained only when alignment quality was high and no evidences of substitution saturation were detected. Phylogenetic trees were computed with the MEGA7 software (Kumar et al., 2016) according to the Maximum likelihood method by applying the best fitting evolutionary model selected by the program itself (i.e. HKY + I + G and HKY + G for the *cox1* and *ITS2* genes, respectively). To assess the statistical support to the tree topologies, 10.000 bootstrap replicates were calculated.

## 3 Results

### 3.1 Morphological description of nematodes collected from *B. physalus*

#### 3.1.1 Adult nematodes

Specimens of *C. boopis* (1 head, 2 male tails and 2 female tails) were deposited at the NHMUK (*Crassicauda boopis* 2015.10.11.1–4).

Morphometric data of adult nematodes isolated from the kidneys of fin whales were in accordance to the description of *C. boopis* in literature (Baylis, 1920; Delyamure, 1955; Lambertsen, 1985).

**Fig. 1:**
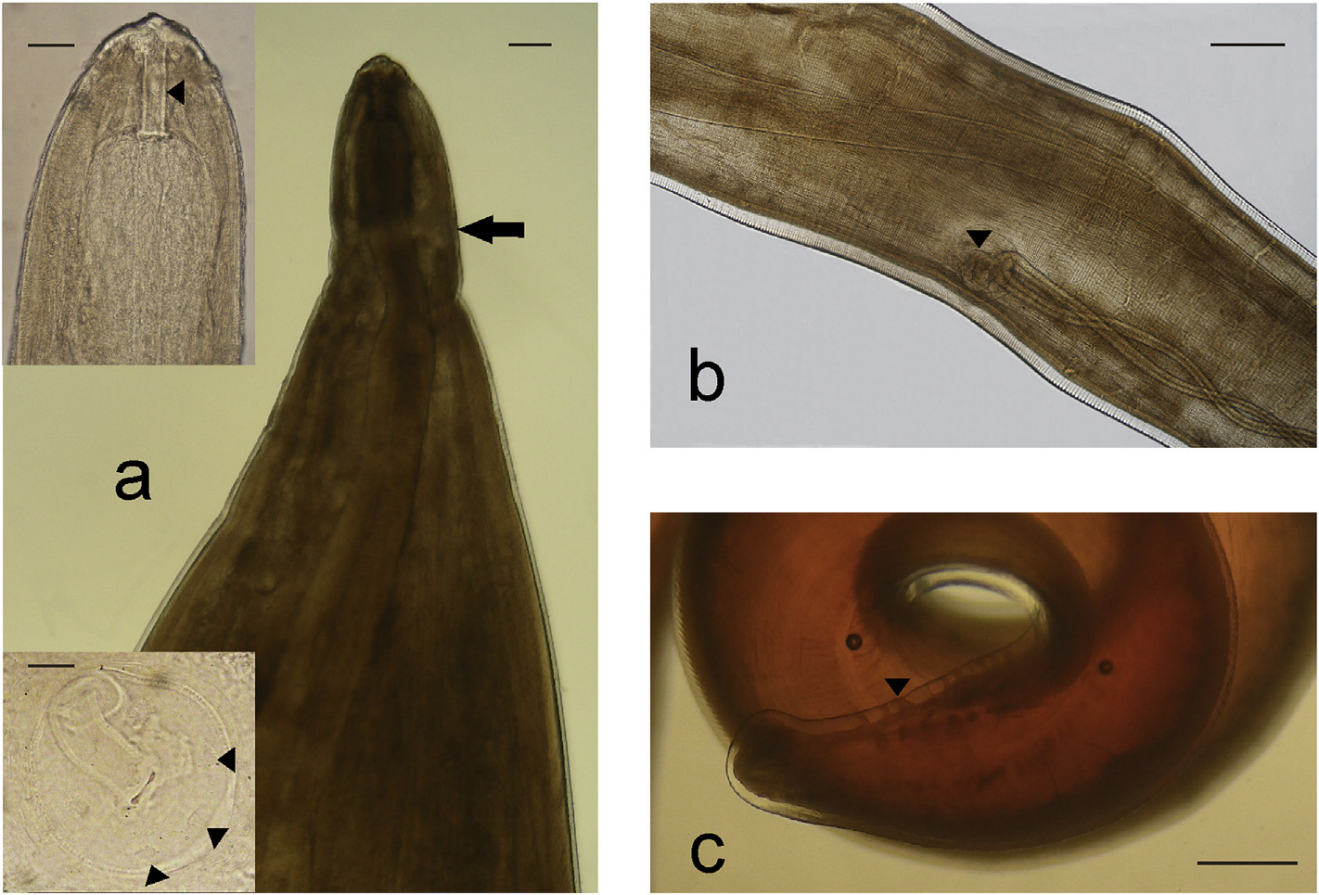
Adult *Crassicauda boopis*. **a**) Female. Cephalic end (bar = 120  μm) with particular of the buccal cavity (arrowhead in the upper inset, lateral view; bar = 60 μm) and of lateral and submedial cephalic papillae situated along the margin of a cuticular plate (arrowheads in lower inset, apical view; bar = 25 μm). **b**) Female. Paired uteri opening in the vulva (arrowhead) (bar = 600  μm). **c**) Male. Coiled caudal extremity with caudal papillae (arrowhead) (stereomicroscope) (bar = 1 mm).

*Female*. Elongated body with maximum length of cephalic fragments of 60.5 cm. The anterior end (measured specimens: n = 2) lying free in the lumen of vena cava, narrowing towards the head, with an evident constriction at 53.5 ± 2.12 μm (range 52–55 μm) from the buccal opening, with a diameter of 90.5 ± 10.5 μm (83–98 μm). The buccal cavity appeared laterally compressed (21.5 × 108 μm); two lips in lateral position, triangular in shape (74 μm wide at the base and 30 μm high), each with a small apical labial papilla. Two lateral and four submedial cephalic papillae were visible on the anterior end (Fig. 1a, lower inset; Fig. 3i). The esophagus was encircled by a nerve ring at 275 ± 0.7 μm (274.0–275.0 μm) from anterior extremity (Fig. 1a) (esophagus length 1200 μm). Paired ovaries and uteri were visible.

The posterior end (measured specimens n = 3), found in the ureters, had a genital constriction at 5600 ± 503 μm (range 5000–6000 μm) from the tip of the tail, where the vulva opened ventrally (Fig. 1b). The pineal appendage contained last part of intestine that ended at 417 ± 38.6 μm (range 375–451) from the terminal end.

*Male*. A cephalic extremity (measured specimens: n = 1) was strongly fixed in the renal parenchyma and the fragment was 8.5 cm long. In the anterior part of the parasite a constriction was observed at about 50 μm from the top, giving the extremity a triangular shape. The mouth was characterized by a slit with two evident triangular lips (88 μm wide at the base and 33 μm high) with two small labial papillae and two well visible lateral cephalic papillae. The buccal cavity appeared laterally compressed (20 × 110 μm).

The posterior end (measured specimens n = 3), found in ureters, was narrowing with a coiled tail (2 turns); the cloaca opened at 1040.6 ± 57.2 μm (999-1106 μm)-from the tip of the tail, with no spicules. Eighteen caudal papillae were visible (Fig. 1c).

#### 3.1.2 Larvae

All measures in this section are average values; please refer to Table 2 for ranges and standard deviation values.

**Table 2:** Mean, standard deviation and range (in brackets) of morphometric data of larval elements (μm). INL, Intestinal Nodules larvae; MAL, Mesenteric Arteries Larvae; FIL, Free Larvae in Intestinal lumen. M = immature male; F = immature female.

	INL	MAL	FIL
Total length	8356.81 ± 679.36 (7201.81–8937.53)	10613.79 ± 1096.75 (9838.27–11389.31)	30417.45 ± 3377.37 (28018.41–34279.7)
Mid body width	81.53 ± 6.81 (69.54–85.04)	107.33 ± 16.90 (91.78–130.23)	151.08 ± 19.09 (124.19–178.00)
Head width	39.29 ± 2.46 (35.25–42.32)	41.49 ± 8.20 (35.68–47.29)	66.05 ± 9.27 (56.54–78.89)
Distance *pseudolabia* – head base	25.69 ± 5.56 (18.9–38.49)	21.73 ± 7.80 (16.21–27.25)	36.08 ± 2.55 (32.06–39.20)
Distance excretory pore-cephalic end	209.46 ± 15.02 (226.79–200.03)	171.72	269.28 ± 19.22 (251.32–299.50)
*Tooth* length	3.24 ± 0.88 (2.51–4.01)	2.78 ± 0.17 (2.65–2.90)	/
Head cuticle thickness	2.08 ± 0.55 (1.08–2.66)	1.44 ± 0.36 (1.19–1.70)	4.42 ± 1.51 (2.31–6.40)
Width of tail at anal opening	61.15 ± 7.82 (47.65–73.99)	64.69 ± 4.79 (64.07–72.93)	99.34 ± 11.84 (88.12–113.59)
Width of tail at genital pore	/	/	154.01 ± 18.28 (139.61–174.58)
Distance anus – terminal end	62.01 ± 6.79 (57.84–71.03)	71.44 ± 5.33 (60.91–72.62)	101.67 ± 4.84 (98.11–107.18) F
Distance cloacal opening/genital pore – terminal end	/	/	141.98 ± 0.88 (141.01–142.75) M 544.11 ± 35.26 (503.63–568.18) F
Cuticle thickness at mid body	5.63 ± 1.90 (4.28–9.74)	5.48 ± 1.69 (3.71–7.09)	7.82 ± 2.51 (4.46–12.56)
Cuticle thickness in terminal end	4.22 ± 1.01 (3.01–5.93)	4.43 ± 1.20 (2.36–4.55)	4.89 ± 1.05 (3.74–5.67)
Length of buccal cavity	/	/	63.91 ± 3.49 (59.4–68.61)
Width of buccal cavity	/	/	15.19 ± 3.67 (10.01–22.41)

**Fig. 2:**
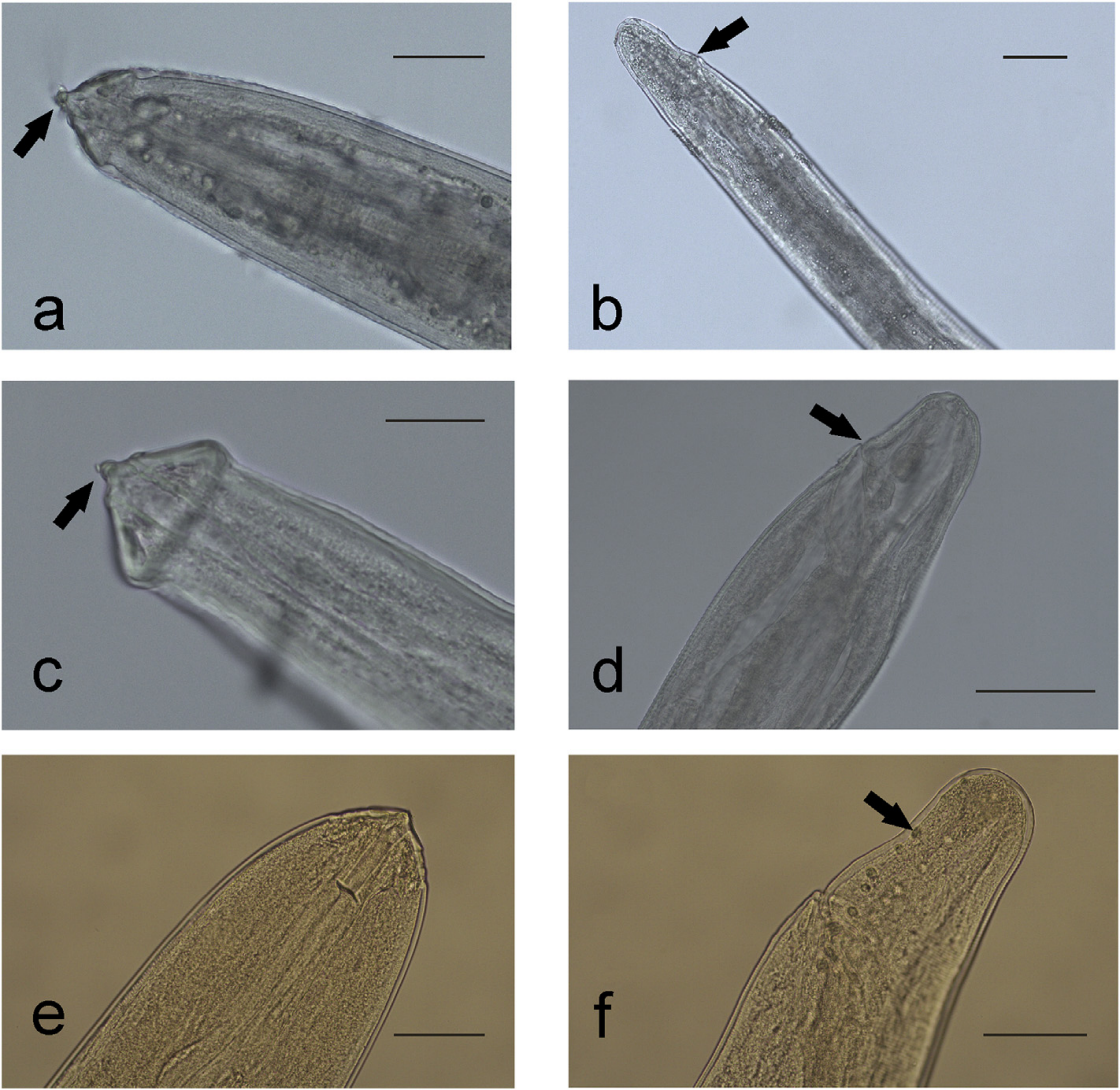
Larval *Crassicauda* spp. **a**, **b**) Cephalic and caudal end of larvae from intestinal nodules (**a**, bar = 40 μm; **b**, bar = 60 μm). **c**, **d**. Cephalic and caudal end of larvae isolated from mesenteric arteries wall (c, bar = 25 μm; d, bar = 65 μm). In **a** and **c** the black arrow indicates lips with terminal tooth, while in **b** and **d** black arrow indicates the anus. **e**, **f**) Cephalic and caudal end of larvae isolated from intestinal lumen, with caudal papillae visible in **f** (black arrow) (**e**, bar = 60 μm; **f**, bar = 70 μm).

**Fig. 3:**
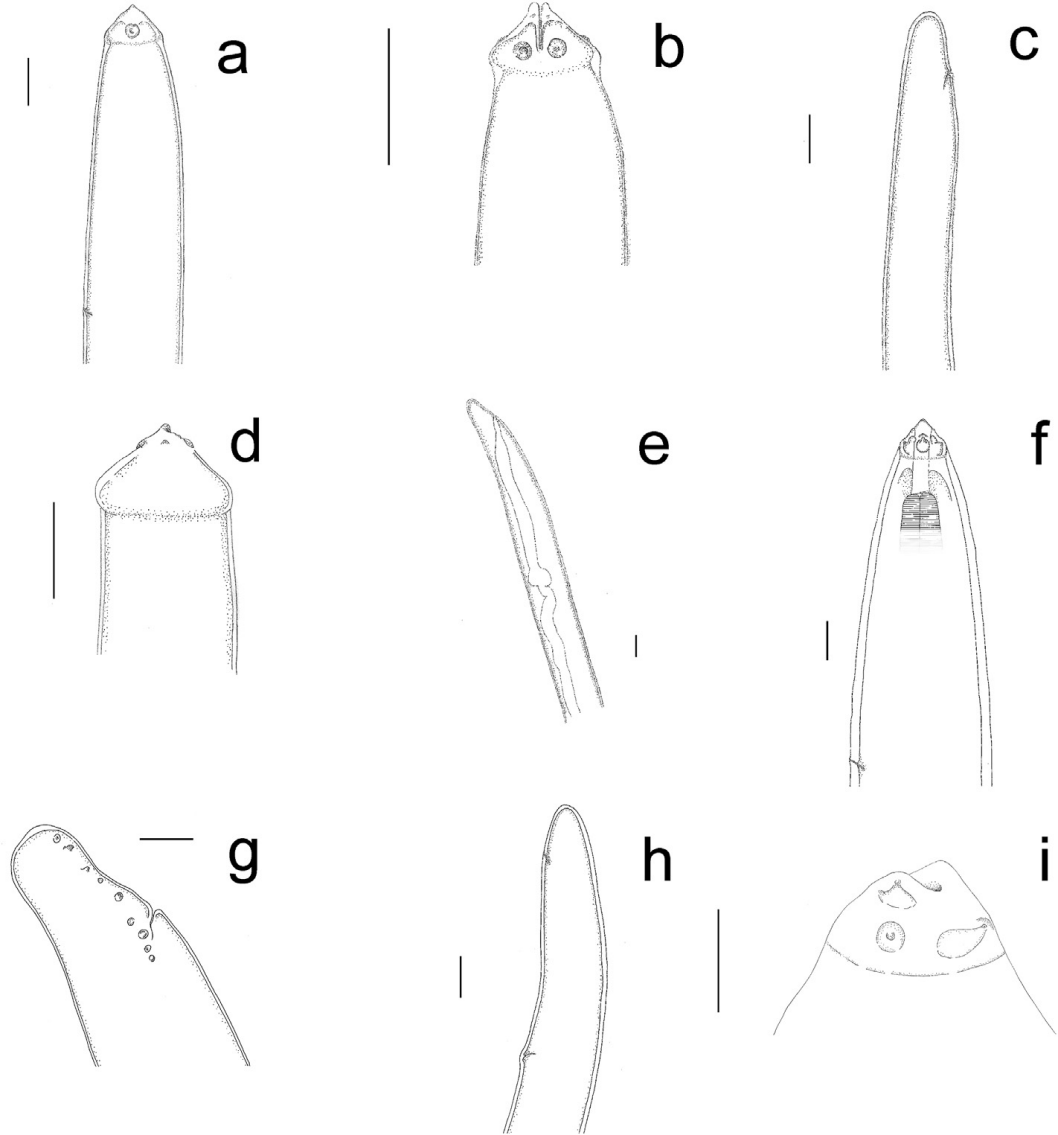
Features of the larvae isolated from fin whales. **a**, **b**, anterior end of larvae included in intestinal nodules, showing cephalic papillae and excretory pore (**a**, lateral view, bar = 35 μm**; b,** dorso-ventral view, bar = 50 μm). **c**, tail of the same larvae, showing the cloacal pore (bar = 60 μm). **d**, anterior end of larvae from the mesenteric arteries, showing bulging of the triangular-shaped head (lateral view, bar = 30 μm); **e**, tail of the same larvae with intestinal tube evident, ending in the cloacal opening (bar = 60 μm). **f**, anterior end of larvae found free within intestinal lumen, showing triangular shape of the anterior region, with cephalic papillae, buccal cavity and excretory pore (lateral view, bar = 25 μm); **g**, **h,** posterior end of the same larvae, showing either presence of a cloaca with multiple papillae (**g,** bar = 50 μm) or a genital pore (**h**, bar = 80 μm); **i**, anterior end of adult *C. boopis*, displaying triangular shaped lips and labial and cephalic papillae (sublateral view, bar = 50 μm).

*Intestinal nodules.* Larvae (n = 7) contained in the intestinal mucosa of one of the adult whales showed the following morphometric features: total length of the body 8356.0 μm, width 81.5 μm. Anterior end was sharp; two lips with terminal rounded tooth, 2 lateral and 4 submedial prominent papillae were observed (Fig. 2a and Fig. 3a and b). Excretory pore was located at 209.5 μm from anterior extremity and anus at 62.0 μm from tip of tail. The posterior end, starting from the cloacal opening, terminated with a rounded tip (Figs. 2b and 3c).

*Mesenteric artery walls.* Larvae were included in the thickness of the vessels’ intimal layer (n = 4). Morphological features were generally similar to those of the larvae included in the intestinal nodules. Total length of the body was 10,613.8 μm, width 107.3 μm. A triangular shaped head, bulging at the base, was observed. Excretory pore at 171.7 μm from anterior extremity (Figs. 2c and 3d). The intestinal tube was evident in central and terminal segment of the body opening in the anus at 71.4 μm from the tip of the tail (Figs. 2d and 3e).

*Intestinal lumen.* Free larvae inside intestinal lumen (n = 9) had a length of 30,417.4 μm and width of 151.1 μm. The anterior end was sharp, with two lips and six cephalic papillae (two lateral and four submedial). The buccal cavity was compressed and measured 63.9 μm × 15.2 μm. Excretory pore was located at 269.3 μm from anterior extremity (Figs. 2e and 3f). In three specimens, the posterior portion had a terminal restriction in which a cloaca with pre and post cloacal papillae (9–10 pairs) was visible at 141.9 μm from the tip of the tail (Figs. 2f and 3g). In other three specimens, a vestigial genital pore and anus were observed at 544.1 μm and 101.7 μm from the tip of the tail respectively (Fig. 3h).

Cuticle transversely striated all along the body length was observed in all larval stages.

### 3.2 Molecular and phylogenetic analysis

Amplification and sequencing of 18S gene resulted in 23 good quality sequences and their alignment (1600 bp long) proved them to be all identical. The Blast research in GeneBank revealed them to be highly similar (99.88%) also to the sequence of *C. magna* (Acc. number KM233410) and *C*. *boopis* (Acc. number KY263809) already deposited. Thus these sequences proved useless to discriminate among different *Crassicauda* species.

**Fig. 4:**
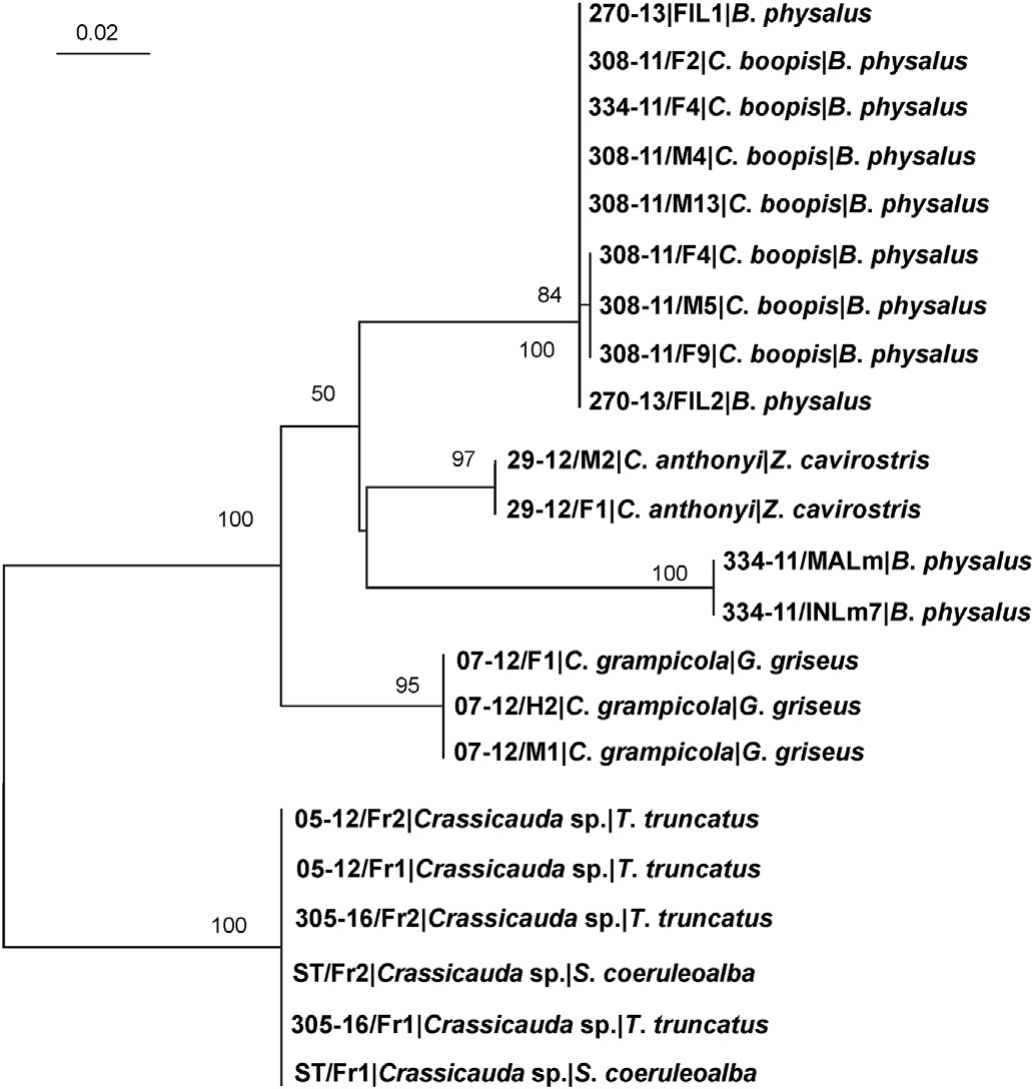
Maximum likelihood tree (Log-likelihood: −1044.368) obtained from ITS2 alignment. The tree was rooted on midpoint. Bootstrap support values (≥50%) are provided near the corresponding node. The scale bar represents 0.02 substitutions/site. Newly determined sequences are in bold.

Amplification of ITS-2 fragment gave 22 sequences (alignment length 435 bp) of good quality (Acc. Numbers MK631888 - MK631909). The specimens, represented by fragments of adult parasites belonging to the species *C*. *boopis* were split in two clusters. All specimens belonging to *C*. *grampicola* exhibited identical sequences. The same evidence was reported for specimens of *C*. *anthonyi*. Specimens, represented by fragments of adult parasites, assigned to *Crassicauda* sp. exhibited a single sequence. Two ITS-2 sequences of the larvae found free in the intestinal lumen (FIL) appeared to be identical to that of *C*. *boopis*. On the other side, larval specimens included in the intestinal nodules (INL) and in the mesenteric artery's wall (MAL) were part of a genetically distant cluster (p-distance ranging between 9 and 9.2%) compared to *C. boopis* cluster. The phylogenetic analyses of all sequenced individuals is provided in Fig. 4.

**Fig. 5:**
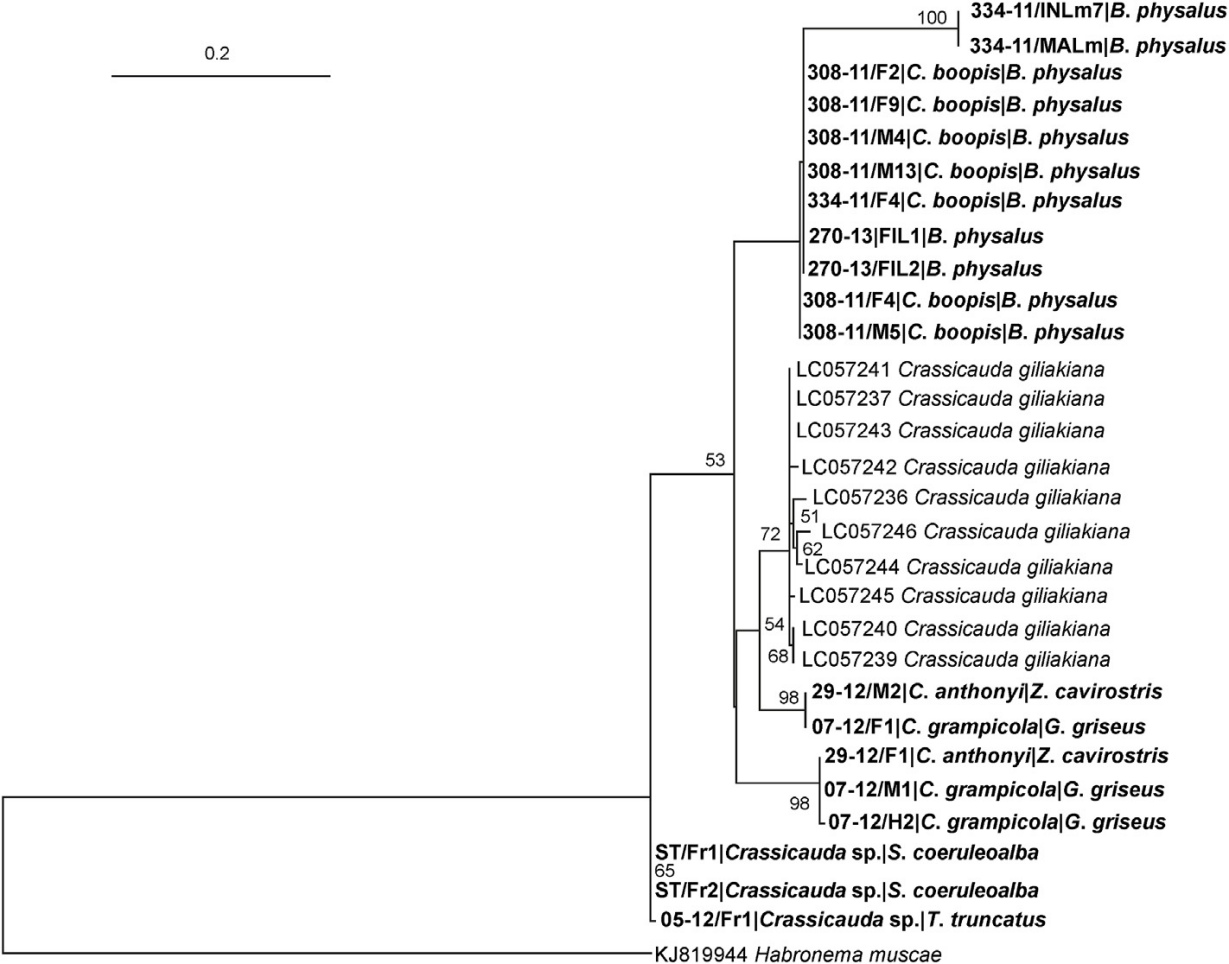
Maximum likelihood tree (Log-likelihood: −1359.826) obtained from *cox1* alignment. The tree was arbitrarily rooted on midpoint. Bootstrap support values (≥50%) are provided near the corresponding node. The scale bar represents 0.2 substitutions/site. Newly determined sequences are in bold.

Amplification of *cox1* gene gave 19 sequences (alignment length 259 bp) of good quality (Acc. Numbers MK621821 - MK621839). The phylogenetic tree based on the *cox1* sequence provided a much complex pattern. The samples of adult *C*. *boopis* and free intestinal larvae were classified in a single cluster. On the other hand, specimens of *C*. *anthonyi* and *C*. *grampicola* were intermingled in two independent clusters comprising substantially identical sequences despite the species difference (Fig. 5). Samples from *Crassicauda* sp. formed a single monophyletic cluster. Two sequences from mesenteric (MALm) and intestinal larvae (INL7) respectively formed, similarly to what observed with the ITS2 gene, an independent group, loosely related (p-distance ranging between 8.2 and 8.5%) to the *C. boopis* one. The analysis of the topology of the obtained *cox1* sequences reveals that, with the exception of adults *Crassicauda* sp. and *C. boopis*, other species of *Crassicauda* do not form monophyletic groups.

Details of all the obtained sequences are reported in Supplementary Table 1.

## 4 Discussion

Fourteen species have been described inside the genus *Crassicauda*, i.e. *C. crassicauda* (Creplin, 1929), *C. giliakiana* Skrjabin and Andreeva, 1934, *C. anthonyi*, *C. bennetti* Spaul, 1926, *C. grampicola*, *C. boopis* (syn. *C. pacifica*), *C. magna* Johnston and Mawson, 1939 (syn. *C. duguyi*), *C. tortilis* Skrjabin (1959), *C. delamureana* Skrjabin, 1966, *C. fuelleborni* Baylis, 1932, *C. costata* Skrjabin (1969), *C. carbonelli* Raga and Balbuena, 1990. These species occur in the kidneys (*C. giliakiana*, *C. anthonyi*, *C. bennetti*, *C. boopis*, *C. tortilis*, *C. delamureana*, *C. costata*), reproductive system (*C. crassicauda*, *C. carbonelli*, *C. fuelleborni*), pterygoid sinuses (*C. grampicola*) and subcutaneous tissues and “gill slit” gland (*C. magna*) of the host (Keenan-Bateman et al., 2018). Among them, four species have been described in the kidneys of mysticetes, i.e. *C. boopis, C. tortilis, C. delamureana, C. costata* showing low host-specificity. The morphological identification of adult specimens is primarily based upon presence of the spicules, which are absent in *C. boopis* and *C. tortilis* and present in *C. delamureana* and *C. costata* (Skrjabin, 1969). *C. boopis* has been isolated in fin whale, blue whale and humpback whale, whereas *C. tortilis* has been described in *Balaenoptera musculus* (Skrjabin, 1973) (Skrjabin, 1959; Lambertsen, 1992). The distinction between the two species relies mainly upon measurements, that are rather similar. In the present study, the isolation of the intact tails and heads of males and females of *C. boopis* allowed the identification of the species in three fin whales, by comparison with the description of the species made by Lambertsen (1985) and Delyamure (1955).

To date, knowledge of the life cycle of *Crassicauda* species. is incomplete, but hypotheses on transmission, migration and development of larvae have been formulated for *C*. *boopis*, by observing pathological findings in infected fin whales (Lambertsen, 1986).

In this study, larval nematodes with morphological features of progressively more advanced developmental stages were isolated from mesenteric arteries, intestinal walls and intestinal lumen of fin whales, respectively. The phylogenetic analysis performed on 18S sequences of these larvae and of all adult nematodes from the different hosts allowed to assign all of them to the genus *Crassicauda*. The 18S marker is a conserved region, which efficiently allows to group deeply phylogenetically separated taxa inside nematode clade. This is confirmed by this study, in which all new sequences obtained from the different species of genus *Crassicauda* were identical to each other, as well as highly similar to *C. magna*, as already evidenced by Dìaz-Delgado et al. (2016) and Lempereur et al. (2017). Analysis of the ITS-2 sequences efficiently grouped together specimens morphologically identified as the same species (i.e. *C*. *grampicola* and *C*. *anthonyi*), as reported for other nematode families (Chilton, 2004; Powers et al., 1997) included the Spirurida (Traversa et al., 2004). Interestingly, the ITS-2 sequences of the well-developed larvae isolated from intestinal lumen of the newborn calf were identical to those from adult *C*. *boopis* collected from the kidneys of adult whales, and this may open new insights on migration and development of *C*. *boopis* larvae. The hypothesis on the migration of *C*. *boopis* larvae indeed suggests the passage of the immature parasites through the gut's mucosal and submucosal layers after ingestion to reach the mesenteric arteries' walls. Still according to the lesions pattern, they finally reach the aorta and renal arteries either by migrating within vessels' walls or by getting into the blood flow (Lambertsen, 1986). In our case, larvae of *C*. *boopis* found within intestinal lumen of the calf, were found to be in an advanced developmental stage; thus, we speculate the migration through the intestinal walls could occur quite late in the developmental process of the nematode. Alternatively, different migration routes could actually exist for *C*. *boopis* in newborn calves, and aberrant migrations cannot be excluded as well. Remarkably, larvae detected in the mesenteric arteries demonstrated quite peculiar genetic features in the ITS2 (p-distance to *C. boopis* = 9%) (and *cox1* gene as well - p-distance to *C. boopis* = 8.2%). If this variability can be explained by a remarkable within-species variability or if it is due to the detection and sequencing of still unclassified *Crassicauda* species remains to be elucidated. Unfortunately, the absence of additional data on ITS-2 sequences from other *Crassicauda* species prevents a further specimen characterization. We speculate that at least two other species should be molecularly characterized to perform comparison with the larvae recovered, i.e. *C. crassicauda* and *C. tortilis*. The first one is the only other species of *Crassicauda* described in fin whales. In the study by Lambertsen (1992), adults of *C*. *crassicauda* were simultaneously present with adult *C*. *boopis* and with *larvae migrans* in the mesenteric arteries; no hypothesis on the life cycle of this species have been formulated. On the other side, *C*. *tortilis* is the only other species described in the kidneys of a mysticete (*Balaenoptera musculus*). Assuming that similar final localization could correspond to similar intra-somatic migration route - as observed for *Crassicauda* sp. in Cuvier's beaked whale's kidneys (Dìaz-Delgado et al., 2016) and considering that the genus *Crassicauda* is characterized by a relatively low host specificity inside the suborders of the odontocetes and mysticetes, a wider molecular assessment inside the genus would be essential to characterize related species and investigate the life cycle with confidence.

Sequencing and phylogenetic analysis of the mitochondrial *cox1* gene revealed a less interpretable pattern, since samples morphologically classified in the same species demonstrated a heterogeneous mitochondrial genetic background and were not monophyletic in the tree. The actual causes of the observed phenomenon remain elusive and further analysis based on more extended dataset should be performed to investigate this issue. Therefore the need for collaborative efforts among research groups and the definition of shared reference genes and regions to be sequenced appears pivotal to understand the evolutionary relationship among *Crassicauda* genus members. Nevertheless, although preliminary, the obtained evidences and especially the comparison between morphological and genetic features, suggests ITS2 to be a more reliable marker for taxa identification within the *Crassicauda* genus.

Overall, the results of the present study emphasize the limits of current classification criteria and the need of a much more extensive and systematic analysis of the morphometric and genetic features of the genus *Crassicauda*, in order to establish an updated, shared and handier classification scheme.

This work contributes both to enrich the genetic sequence data bank and the molecular taxonomy of this genus; it represents an important starting point for identification of ruined fragments or immature stages of *Crassicauda* spp.
