# Supplementary material for: Morphological and molecular characterization of adults and larvae of Crassicauda spp. (Nematoda: Spirurida) from Mediterranean fin whales Balaenoptera physalus (Linnaeus, 1758)
Source: Int J Parasitol Parasites Wildl. 2019 Jun 7;9:258–65. doi: 10.1016/j.ijppaw.2019.06.004 (PMC6580233; doi:10.1016/j.ijppaw.2019.06.004)
Supplement: Multimedia component 1 [file mmc1.docx]

| **Host** | | |  | **ITS-2** | |  | ***cox 1*** | |
| --- | --- | --- | --- | --- | --- | --- | --- | --- |
| **Species** | **Year of stranding** | **Site** | **Species** | **Specimen** | **Accession number** |  | **Specimen** | **Accession number** |
| *Balaenoptera physalus* | 2011 | Thyrrenian Sea | *Crassicauda boopis*  (Localization: kidney) | 308-11 M13 | MK631897 |  | 308-11 M13 | MK621830 |
|  |  |  |  | 308-11 M4 | MK631895 |  | 308-11 M4 | MK621828 |
|  |  |  |  | 308-11 F2 | MK631892 |  | 308-11 F2 | MK621825 |
|  |  |  |  | 308-11 F4 | MK631893 |  | 308-11 F4 | MK621826 |
|  |  |  |  | 308-11 F9 | MK631894 |  | 308-11 F9 | MK621827 |
|  |  |  |  | 308-11 M5 | MK631896 |  | 308-11 M5 | MK621829 |
| *Balaenoptera physalus* | 2011 | Thyrrenian Sea | *Crassicauda boopis*  (Localization: kidney) | 334-11 F4 | MK631898 |  | 334-11 F4 | MK621831 |
|  |  |  | *Crassicauda* sp. larvae (Localization: mesenteric arteries) | 334-11 MALm | MK631900 |  | 334-11 MALm | MK621833 |
|  |  |  | *Crassicauda* sp. larvae (Localization: intestinal nodules) | 334-11 INLm7 | MK631899 |  | 334-11 INLm7 | MK621832 |
| *Balaenoptera physalus* | 2013 | Thyrrenian Sea | *Crassicauda* sp. larvae (Localization: intestinal lumen) | 270-13 FIL 1 | MK631890 |  | 270-13 FIL 1 | MK621823 |
|  |  |  |  | 270-13 FIL 2 | MK631891 |  | 270-13 FIL 2 | MK621824 |
| *Grampus griseus* | 2012 | Adriatic Sea | *Crassicauda grampicola*  (Localization: cranial sinuses) | 07-12 H2 | MK631901 |  | 07-12 H2 | MK621836 |
|  |  |  |  | 07-12 F1 | MK631903 |  | 07-12 F1 | MK621834 |
|  |  |  |  | 07-12 M1 | MK631902 |  | 07-12 M1 | MK621835 |
| *Stenella coeruleoalba* | 2012 | Adriatic Sea | *Crassicauda* sp.  (Localization: subcutaneous tissues) | 05-12 Fr1 | MK631904 |  | 05-12 Fr1 | MK621837 |
|  |  |  |  | 05-12 Fr2 | MK631905 |  |  |  |
| *Tursiops truncatus* | 2006 | Adriatic Sea | *Crassicauda* sp.  (Localization: subcutaneous tissues) | 305-06 Fr1 | MK631906 |  |  |  |
|  |  |  |  | 305-06 Fr2 | MK631907 |  |  |  |
| *Tursiops truncatus* | 2014 | Thyrrenian Sea | *Crassicauda* sp.  (Localization: subcutaneous tissues) | ST Fr1 | MK631908 |  | ST Fr1 | MK621838 |
|  |  |  |  | ST Fr2 | MK631909 |  | ST Fr2 | MK621839 |
| *Ziphius cavirostris* | 2012 | Ionian Sea | *Crassicauda* *anthonyi*  (Localization: kidney) | 29-12 F1 | MK631888 |  | 29-12 F1 | MK621821 |
|  |  |  |  | 29-12 M2 | MK631889 |  | 29-12 M2 | MK621822 |

**Supplementary table 1**. Details of cetaceans positive to *Crassicauda* spp. and accession numbers of the parasite specimens morphologically and molecularly analyzed in this study.
